# A Qualitative Study on Making Rural Communities More Dementia-Friendly

**DOI:** 10.1093/geroni/igaa057.899

**Published:** 2020-12-16

**Authors:** Melissa OConnor, Megan Pedersen, Rachel Grace

**Affiliations:** North Dakota State University, Fargo, North Dakota, United States

## Abstract

Recent studies on attitudes toward dementia in the United States, such as the World Alzheimer Report 2019, have found that fear and stigma are still widespread among the general public. This may be particularly true in rural communities. In the current study, community-dwelling adults in small Midwestern communities responded to the open-ended survey question, “What do you think could be done to make your community more welcoming for people with Alzheimer’s disease and other forms of dementia?” Participants (N=242) ranged in age from 18-88 (M=40, SD=21). The sample was 68% female, and 61% lived in communities of 50,000-150,000 people, while 39% lived in smaller towns. Most participants (61.2%) did not personally know someone with dementia. Data were collected via paper and telephone surveys. Responses to the open-ended question were analyzed using open, axial, and selective coding. The following themes emerged: greater exposure to individuals with dementia; educational workshops about dementia; more intergenerational programs; greater accessibility of respite care and other services; more fundraising efforts; and community leaders talking about dementia. Responses included, “Have more intergenerational programs that bring together Alzheimer’s patients and children in a positive environment.” “When I was in school, we visited an Alzheimer’s unit. That was a great experience.” “I know what it is, but I don’t know anything else. I wish I was more informed. I don’t know how to help.” These findings indicate that residents of rural communities are motivated to help individuals with dementia, but need more guidance, education, and personal connections/exposure.

